# Goos-Hänchen effect in epsilon-near-zero metamaterials

**DOI:** 10.1038/srep08681

**Published:** 2015-03-03

**Authors:** Yadong Xu, C. T. Chan, Huanyang Chen

**Affiliations:** 1 College of Physics, Optoelectronics and Energy, Soochow University, No.1 Shizi Street, Suzhou 215006, China; 2 Department of Physics and Institute for Advanced Study, Hong Kong University of Science and Technology, Clear Water Bay, Hong Kong, China

## Abstract

Light reflection and refraction at an interface between two homogeneous media is analytically described by Snell's law. For a beam with a finite waist, it turns out that the reflected wave experiences a lateral displacement from its position predicted by geometric optics. Such Goos-Hänchen (G-H) effect has been extensively investigated among all kinds of optical media, such as dielectrics, metals, photonic crystals and metamaterials. As a fundamental physics phenomenon, the G-H effect has been extended to acoustics and quantum mechanics. Here we report the unusual G-H effect in zero index metamaterials. We show that when linearly polarized light is obliquely incident from air to epsilon-near-zero metamaterials, no G-H effect could be observed for *p* polarized light. While for *s* polarization, the G-H shift is a constant value for any incident angle.

It is well known that when light is totally reflected from an interface of two media, it will experience a lateral displacement from the position predicted by the geometric optics. Such a lateral displacement is called the Goos-Hänchen (G-H) shift^1^. In the past few decades, the G-H effect has been extensively explored in numerous interfaces involving absorptive dielectrics^2^,^3^,^4^, metals^5^,^6^ and photonic crystals^7^,^8^,^9^. Particularly, the total reflected wave undergoes a negative G-H shift for a metamaterial with negative refractive index^10^. Apart from total reflections, the G-H effect has also been observed for partial reflections or transmissions at the interfaces^4^,^11^. Due to its broad interest, the G-H effect has also been extended to other physics systems, including nonlinear optics^12^, acoustics^13^ and quantum mechanics^14^,^15^. Several theoretical methods have been developed to understand the G-H effect, such as stationary phase and energy propagation^16^,^17^. For the stationary phase method, the G-H shift can be given by
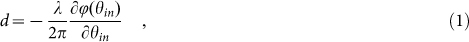
where *λ* is the wavelength, *ϕ*(*θ_in_*) is the phase of the reflected coefficient and *θ_in_* is the incident angle.

Recently, zero index metamaterials (ZIMs) have drawn much attention for their intriguing electromagnetic (EM) properties^18^,^19^,^20^,^21^,^22^,^23^,^24^,^25^,^26^,^27^. In particular, as a kind of ZIMs, the epsilon-near-zero (ENZ) metamaterials has attracted more attention because they can be found in nature or be fabricated easily, *e.g*, the plasmonic materials at optical frequency^28^ or doped semiconductors at terahertz (THz) region^29^. Nevertheless, to the best of our knowledge, there is no research on the G-H effect in ZIMs. Generally, the G-H shifts are positive among lossless dielectrics with a positive index, while they turn negative in lossless metamaterials with a negative index. It seems that the G-H shifts should be zero in ZIMs. In this work, we will explore the G-H effect when a linearly polarized light is obliquely incident from air onto an ENZ metamaterial. We decompose the linearly polarized light into two independent polarizations, *i.e.* the *s* polarized light with its electric-field vector perpendicular to the incident plane, and the *p* polarized light with its electric-field vector parallel to the incident plane. It is shown that there is no G-H effect for *p* polarized light, while for *s* polarization, the G-H shift is a constant value, independent of the incident angle. Such a unique behavior has not yet been revealed in previous study on the G-H effect.

## Results

### Theoretical analysis

**Figure 1:**
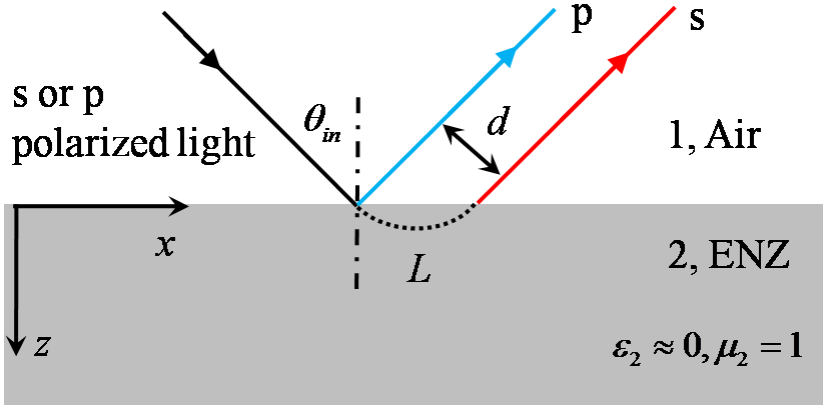
The schematic diagram of G-H effect for a linearly polarized light totally reflected at an interface. Region 1 and 2 are air and ENZ metamaterial, respectively. If the obliquely incident light from air is of p polarization, there is no G-H shift for the reflected light, as shown by the blue line. If the incident light is of s polarization, the G-H shift d is a constant value for any incident angle, as shown by the red line, and the lateral shift is indicated by L. Region 1 and 2 are infinitely extended in x and y directions.

Our configuration is shown the schematically in Fig. 1. An interface in *x*-y plane separates two semi-infinite media. Region 1 is air, and region 2 is ENZ metamaterial with parameters *μ*_2_ = 1 and *ε*_2_ ≈ 0 (we assume that the ENZ metamaterial is homogenous and isotropic). A linearly polarized light with incident angle *θ_in_*is incident from air onto ENZ metamaterial. As known, the ENZ metamaterial can be regarded as optically thinner medium compared to air, and the critical angle for total reflection to occur is given by 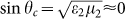. As the permittivity of ENZ metamaterial is about zero, the critical angle tends to zero. Physically, the linearly polarized light, both *s* and *p* polarizations, will be totally reflected by the ENZ metamaterial for any incident angles excluding the normal one.

For G-H effect, however, both polarizations have different responses during such a total reflection process. First, let us consider the *s* polarization. Suppose the incident linearly polarized light with an angular frequency *ω* is given as 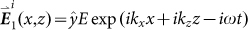, where *k_x_* = *k*_0_ sin *θ_in_*, *k_z_* = *k*_0_ cos *θ_in_*, and *k*_0_ = *ω*/*c* is the wave number in air, *c* is the velocity of light in air. For simplicity, the time dependence exp(−*iωt*) will be omitted throughout this work. After reflection by ENZ metamaterial, the reflected light is 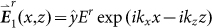. Inside region 2 of ENZ metamaterial, the refracted light is expressed as 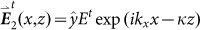, where 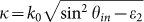. In addition, each corresponding magnetic field can be calculated from one of Maxwell's equations 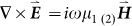. Applying the continuous boundary conditions of tangential electric and magnetic fields at *z* = 0, the reflected coefficient is calculated as 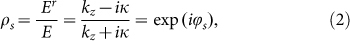
with its phase, 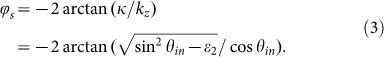
By employing the method of stationary phase as shown by the equation (1), the G-H shift can be achieved as
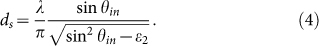
It is noted that this result is not valid at 90°, as the equation (1) for quantitatively analyzing the G-H shift is not defined there^10^. From equation (4), it is obviously seen that when the permittivity *ε*_2_ is close to zero, *i.e.* *ε*_2_ ≈ 0, the G-H shift is *d_s_* = *λ*/*π*, a constant value for any incident angle. Meanwhile, we can work out that the phase of reflected light is *ϕ_s_* = −2*θ_in_* from equation (2), which is a linear function of the incident angle.

A similar procedure can be implemented for *p* polarized light. In this case, electric-field in *y* direction in the above equations should be changed into magnetic field. In region 1 of air, the incident and reflected light can be written as 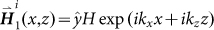 and 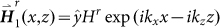, respectively. In the region of ENZ metamaterial, the refracted light is 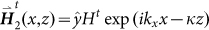. Similarly, the corresponding electric fields could be obtained by one of the Maxwell's equations 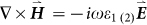. Applying the continuous boundary conditions, the reflected coefficient is calculated as 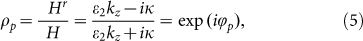
with its phase expressed as
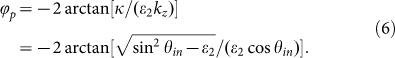
Applying the same method of equation (1), the G-H shift for *p* polarized light is given as 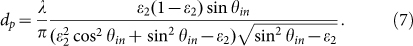
From this equation (7), we know that for the ENZ metamaterial with the permittivity *ε*_2_ ≈ 0, the G-H shift is *d_p_* = 0 for any incident angle. From equation (6), the phase of reflected light is −*π*, which means there is a half-wave phase lag for *p* polarized light after total reflection. Such a feature is analogous to the propagation of the *s* polarized light reflected by the perfect electric metal. With that in mind, the ENZ metamaterial could be regarded as a perfect magnetic conductor (PMC) for *p* polarized light. From the perspective of intrinsic property of ENZ metamaterial, we know that as the permittivity *ε*_2_ tends to zero, the right term 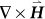 in Maxwell's Equation 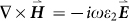 should vanish, in order to keep a finite value of electric field. As a result, the magnetic field inside the ENZ material should be a constant^25^, that is *H*_2_ = *Const*. Then, following the continuous conditions of the magnetic fields at the boundary *z* = 0, we get (*H^r^* + *H*)exp(*ik_x_x*) = *Const*. For the *p* polarized light obliquely incident onto the ENZ material, we have *k_x_* ≠ 0. To keep the above boundary condition valid for arbitrary coordinate *x*, the only satisfaction is that *H^r^* + *H* = 0. It also means that the magnetic field inside ENZ metamaterial is exactly zero. Consequently, the reflected coefficient is *ρ_p_ = H^r^*/*H* = −1 and its reflected phase is −*π*. Such understanding from the intrinsic property itself is more straightforward, and gives the same results on G-H effect for *p* polarized light.

The G-H effect can also be interpreted from the point of view of an energy flow along the interface in the optically thinner medium^16^ (*e.g.* the ENZ metamaterial). The time averaged energy flow can be calculated by applying 
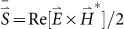
 (the star sign represents the conjugate operation). For *s* polarized incident light, we have 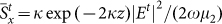 and 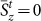. Moreover, the penetrated length could be defined by *l* = 1/*κ*. It implies that the incident EM energy will enter into ENZ metamaterial, and the energy will go back into air when it arrives at *z* = *l*. Moreover, the G-H shift from the view of geometric optics, is *d* ≈ 2*l* sin *θ_in_* = *λ*/*π* when *ε*_2_ ≈ 0. For *p* polarized light, however, the corresponding averaged energy flow is 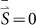 because the magnetic field inside ENZ material is exactly zero. It means that no electromagnetic energy enters into ENZ metamaterial, thereby leading to no G-H effect. This view from the surface energy flow further corroborated our analysis.

### Influence of losses

We considered a lossless ENZ metamaterial in above theoretical analysis. However, for a real ENZ metamaterial, such as the noble metal at plasma frequency or doped semiconductors at terahertz^28^,^29^, their losses should be considered. In the following, we will illustrate the influence of loss on the G-H shifts for *s* and *p* polarized light. For an absorptive medium, the equation (1) quantifying the G-H shift should be rewritten as 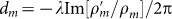 (*m* = *s* and *p*)^30^, where the prime indicates the derivative with respect to *θ_in_*. Combining equation (2) and equation (5), the G-H shifts for two polarizations are given as,
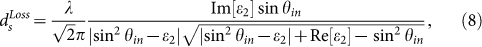

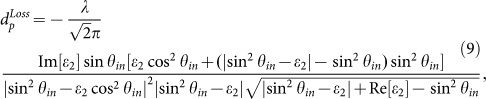
when Im[*ε*_2_] ≠ 0, where Re[*ε*_2_] and Im[*ε*_2_] represent, respectively, the real part and imaginary part of the permittivity of loss ENZ metamaterial. As usual, the imaginary part indicates the loss.

**Figure 2:**
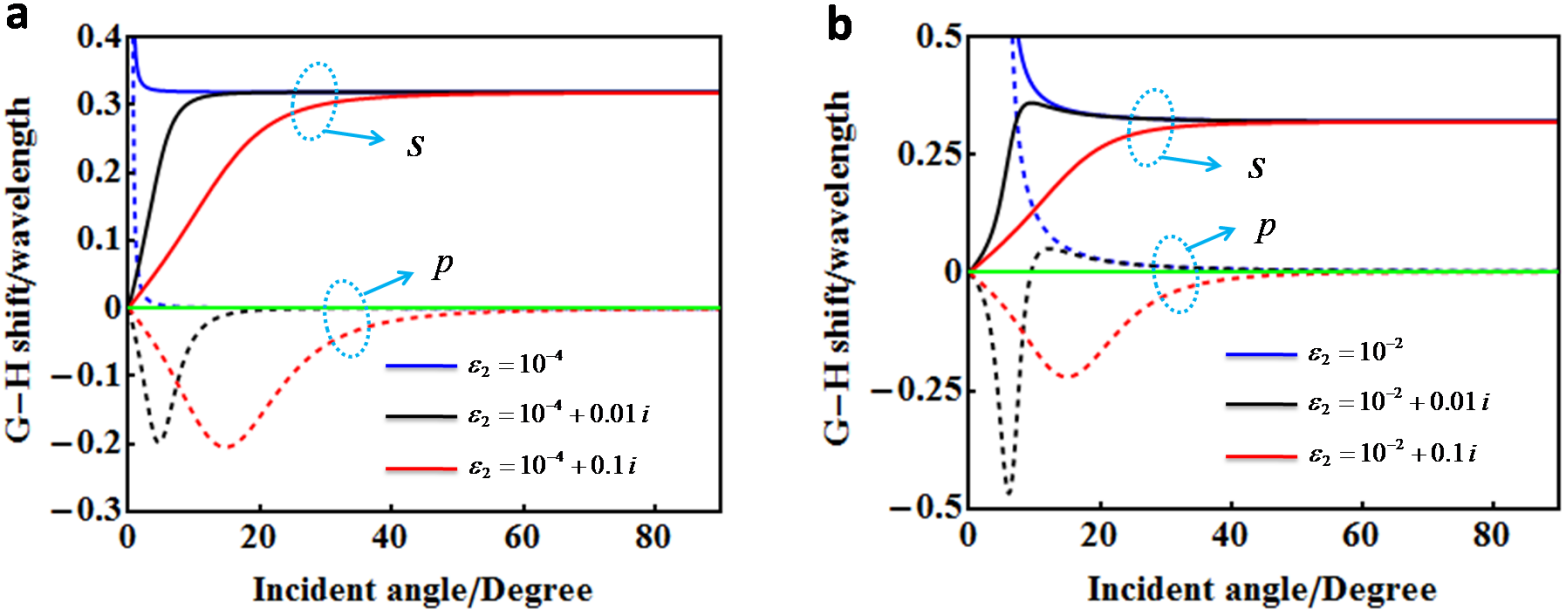
The G - H shifts for ENZ metamaterials with different losses. (a), The blue, black and red curves corresponds to permittivities with *ε_2_* = 10^−4^, 10^−4^+0.01*i* and 10^−4^+0.1*i*, respectively. The solid lines are for s polarization, and the dashed lines are for p polarization. (b), It is the same as (a), but with the permittivities replaced by *ε_2_* = 10^−2^, 10^−2^+0.01*i* and 10^−2^+0.1*i*, respectively.

Based on the above formulas, Fig. 2 shows the calculated G-H shifts for different values of the loss parameter of ENZ metamaterials. In Fig. 2a, we set Re[*ε*_2_] = 10^−4^, and the solid and dashed lines are corresponding to *s* and *p* polarizations, respectively. As shown by the blue lines, the G-H shifts for both polarized light of *s* and *p* are *d_s_* = *λ*/*π* and *d_p_* = 0, respectively, for any incident angle except the vicinity of the critical angle. It is noted that when the incident angles vary across the critical one, the reflection phase will experience an abrupt change, usually leading to quite an obvious G-H effect^31^. When the loss is taken into account, *e.g.* Im[*ε*_2_] = 0.01, for *s* polarized light, as shown by the black solid line, there is a region where the G-H shift gradually changes from zero to *λ*/*π*. Such a property stems from the fact that the added losses make the reflection phases deviate from the linear relationship of *ϕ_s_* = −2*θ_in_*. In particular, the deviation is quite notable at small incident angle. For *p* polarized light, however, the situation is more complicated. Generally, for an absorptive medium, there is an abrupt change of phase across the (pseudo) Brewster angle when a *p* polarized light is reflected by it. As a result, such an abruptly changed phase induces a negative G-H shift around the (pseudo) Brewster angle^3^. It is also true for lossy ENZ metamaterials. For instance, when the loss is Im[*ε*_2_] = 0.01, as shown by the black dashed line, a resonance for G-H shift could be observed. Further, we use a larger loss with Im[*ε*_2_] = 0.1, as shown by the red lines. The gradually changing region of G-H shift for *s* polarized light is enlarged (see the red solid line), while for *p* polarized light, the resonance position is shifted to a larger angle (see the red dashed line). Nevertheless, for an incident angle large enough, the G-H shifts for the loss cases will approach those of the situation without loss, *i.e.*, for *s* polarized light, it is almost *λ*/*π*, while for *p* polarized light, it is close to zero. The larger the loss is, the narrower the range of the working incident angle is. In particular, the working incident angle range for Im[*ε*_2_] = 0.01 is at about [15°, 90°), while for Im[*ε*_2_] = 0.1, it changes into [40°, 90°).

In order to examine the sensitivity of the small values of Re[*ε*_2_] to the G-H shift, Fig. 2b shows the calculated results when Re[*ε*_2_] increases to 10^−2^. For the lossless case, the critical angle becomes larger, then the working angle for both polarizations is accordingly shifted to a larger angle, as shown by the blue lines. When the same levels of losses are involved, the black and red lines are corresponding to those of Im[*ε*_2_] = 0.01 and 0.1, respectively. Similar results can be observed including the gradually changed regions of G-H shifts for *s* polarized light and the resonances of G-H shifts for *p* polarized light. Although the real part of the permittivity is larger, the working incident angle ranges do not vary that much if we compare Fig. 2a and Fig. 2b. For the same loss, the positions of resonances are shifted very slightly when the Re[*ε*_2_] increases from 10^−4^ to 10^−2^. However, the amplitudes of resonances change a little bit. Generally, for an absorptive dielectric, the amplitudes of resonances decrease as the ratio of Im[*ε*_2_]/Re[*ε*_2_] increases^30^. For the ENZ metamaterials with different losses, the amplitudes of resonances have the same tendency.

### Numerical simulations

**Figure 3:**
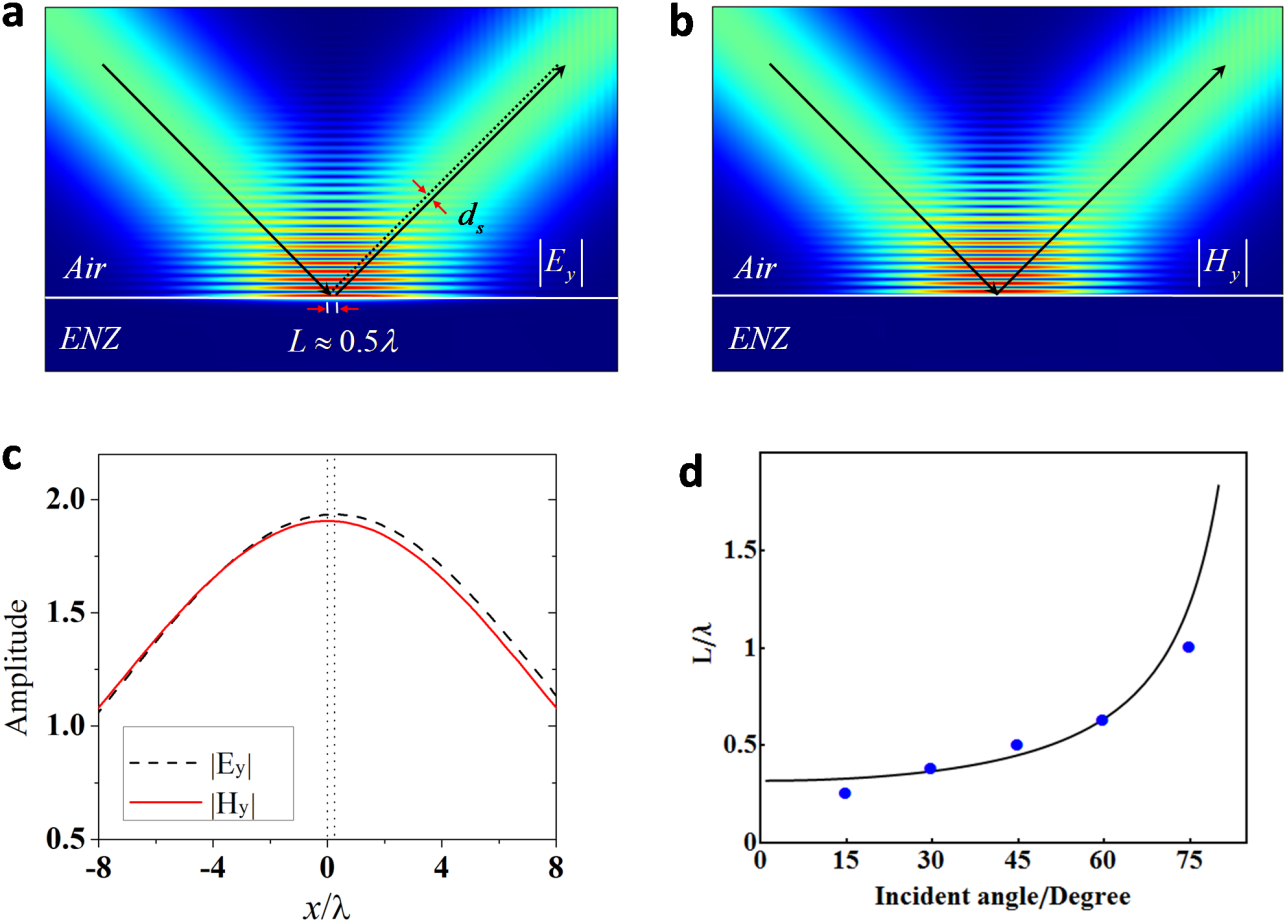
Numercial simulations of G-H shifts when the Gaussian beam is incident from air. (a), The field pattern for s polarization with an incident angle of 45°. (b), The field pattern for p polarization with the an incident angle of 45°. (c), The distributions of field amplitudes along x direction near the interface between ENZ metamaterial and air, based on numerical results in (a) and (b). The red line is for p polarization, and the black dashed line is for s polarization. The two dotted lines indicate the symmetrical axes of each distribution. (d), The comparison for the lateral shift L is based on theoretical analysis (black line) and numerical simulations (blue dots). In all numerical simulations, the permittivity of ENZ metamaterial is set as 10^−4^.

To give a comprehensive understanding and to verify the above theoretical analysis, we perform the numerical simulations of G-H shifts, where we consider a beam with a spatial Gaussian profile incident from air to ENZ metamaterial, and the half-width of the beam is 7.5*λ*. Because of the small G-H shift, it is easier to observe the lateral shift *L* = *d_s_*/cos(*θ_in_*). Fig. 3 shows the simulated results when the incident angle is 45°, in which for *s* polarization, the lateral shift *L* is about 0.5*λ*, as shown in Fig. 3a, while for *p* polarization, the shift is zero, as shown in Fig. 3b. Theoretically, the value is about 0.45*λ* for *s*-polarization. Considering the fact that the incident beam is not a plane wave, such a deviation is acceptable. Moreover, Fig. 3c shows the distributions of field amplitude near the interfaces for two polarizations, as shown by the red line and the black dashed line respectively. It is clearly seen that for *p* polarization, the symmetry axis of the field distribution (the red line) is located at the original point *x* = 0, as shown by the dotted line. While for *s* polarization its symmetric center is shifted to the left. Such a contrast further verifies our theoretical prediction that the G-H shift exists only for *s* polarization. Lastly, we compare the numerical results *L* for different incident angles with the analyzed results, as shown in Fig. 3d. The black line is given by the formula *L* = *d_s_*/cos(*θ_in_*), while the five blue points are the numerical results for incident angles with 15°, 30°, 45°, 60° and 75°, respectively. In the plot, the numerical results fit well with the analytical curve.

Finally, it is worth mentioning that we only focus on the ENZ metamaterials here. In fact, the zero index metamaetrials can be further categorized, *e.g.*, mu-near-zero (MNZ) metamaterials, matched impedance zero-index metamaterials (MIZIMs), and anisotropic ZIMs. For MNZ metamaterials, we predict based on current results that the G-H shift is a constant for *p* polarized light, independent of the incident angle. Yet, there is no G-H shift for *s* polarization. For MIZIMs, there are no G-H shifts for both polarizations of *s* and *p* because the oblique incident light cannot enter into MIZIMs at all.

## Discussion

We have investigated the G-H effect of reflected light, when a linearly polarized light of either *s* or *p* polarization is obliquely incident onto the ENZ metamaterial from air. We have shown that for lossless ENZ material, there is no G-H shift for *p* polarization, which indicates that it obeys exactly the prediction by the geometrical optics. While for *s* polarization, the G-H shift is a constant value, independent of the incident angle. We also have explored analytically the influence of loss of ENZ metamaterials on the G-H shift for both polarizations and we found that the novel features still hold for a broad range of angles. Analogous deviations of G-H effect are expected for acoustic waves, quantum matter waves and so on. In addition, we have shown that the reflected phase of *p* polarized light is *ϕ_p_* = −*π*, while for *p* polarized light, the reflected phase obeys that linear relationship *ϕ_s_* = −2*θ_in_*. Due to such different responses, we believe that the ENZ metamaterial can be utilized to manipulate the polarization states of reflected light, by only adjusting the incident angle. We expect our work to contribute some new notions to the manipulation of the polarization states of light at the THz region.

## Methods

### Theory and simulations

The numerical simulation results shown in Fig. 3a and 3b were obtained using the finite element solver COMSOL Multiphysics. The scattering boundaries were set for four sides. Based on these numerical simulations, the curves of field amplitudes in Fig. 3c were obtained by performing the line plot along *x* axis from −8*λ* to 8*λ*. Due to the interference effect, the field amplitudes are oscillating along *z* direction. The line plots are located at the first peak close to the interface between air and ENZ metamaterial. Meanwhile, we zoom in the line plot of |*E_y_*| enough to get the distance between its symmetric axis and *x* = 0, which indicates the lateral shift *L*. The numerical results in Fig. 3d were obtained by the same technique.

## Author Contributions

Y.X. and H.C. conceived the idea. Y.X. did the theoretical calculations and the numerical simulations. H.C. and C.T.C. helped with the theoretical analysis. H.C. supervised the whole project. All the authors wrote the manuscript.
